# Natural Flavonoids from Licorice as Potent Inhibitors of β-Glucuronidase Elucidated Through Computational Studies

**DOI:** 10.3390/molecules30061324

**Published:** 2025-03-15

**Authors:** Jingli Liu, Yingying Xue, Hao Yan, Jing Zhou, Xu Long, Yuping Tang

**Affiliations:** 1 College of Pharmacy, Shaanxi University of Chinese Medicine, Xianyang 712046, China; liujl@sntcm.edu.cn (J.L.); xueyingying0708@163.com (Y.X.); yanhao@sntcm.edu.cn (H.Y.); 2051124@sntcm.edu.cn (J.Z.); longxu@sntcm.edu.cn (X.L.); 2 Key Laboratory of Shaanxi Administration of Traditional Chinese Medicine for TCM Compatibility, Shaanxi University of Chinese Medicine, Xianyang 712046, China

**Keywords:** licorice flavonoids, inhibitors, β-glucuronidase, molecular docking, molecular dynamics simulation, free energy calculation

## Abstract

Gut bacterial β-glucuronidase is an important molecular target in several therapeutic applications. β-glucuronidase inhibitors can effectively alleviate gastrointestinal toxicity caused by certain drugs. Licorice, a traditional Chinese medicine, harmonizes various herbs and mitigates the toxicity of hundreds of herbs. In this study, a comprehensive computational strategy was employed to evaluate four licorice flavonoids (liquiritigenin, isoliquiritigenin, liquiritin, and isoliquiritin) as potential *Escherichia coli* β-glucuronidase (EcGUS) inhibitors. Density functional theory was used to determine their geometries, thermal parameters, dipole moments, polarizabilities, and molecular electrostatic potentials. The inhibitory mechanisms of these four flavonoids on EcGUS were investigated using molecular docking, molecular dynamics simulations, and free energy calculations. The results show that all four flavonoids stably bind to EcGUS. Moreover, all molecules, except liquiritigenin, are potent and selective inhibitors of EcGUS. Further calculations suggest that isoliquiritin exhibits the strongest binding affinity for EcGUS among the four licorice flavonoids. Thus, isoliquiritin is a promising candidate for the development of EcGUS inhibitors. These findings will aid in designing and developing novel flavonoid-based inhibitors of EcGUS to alleviate gastrointestinal toxicity caused by drugs.

## 1. Introduction

Irinotecan (CPT-11) is a first-line drug used to treat colorectal cancer and other solid tumors. It is a classic inhibitor of DNA topoisomerases, and its primary side effects include delayed diarrhea, nausea, and vomiting. Gastrointestinal (GI) toxicity significantly affects the efficacy of CPT-11 and severely limits its clinical application [1]. According to the pharmacokinetic mechanism [2], CPT-11 is converted to its active and toxic metabolite, 7-ethyl-10-hydroxycamptothecin (SN-38), by carboxylesterase in the liver. After exerting its anti-cancer effects, SN-38 is further metabolized by uridine diphosphate glucuronate transferase 1A1 into its inactive form, SN-38-glucuronide (SN-38G). However, gut bacterial β-glucuronidase (GUS) in the GI tract can hydrolyze SN-38G back into SN-38, thus leading to the GI toxicity of CPT-11. Consequently, gut bacterial GUS has emerged as a key molecular target for alleviating the GI toxicity caused by CPT-11.

**Figure 1:**
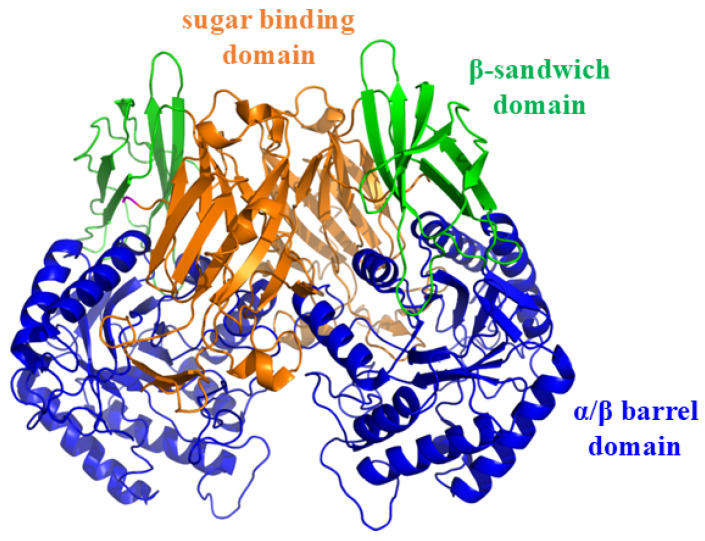
Crystal structure of the EcGUS dimer (PDB:3K46) [5].

GUS (EC 3.2.1.31) belongs to glycosyl hydrolase (GH) families 1, 2, 30, 79, and 154, which are widely distributed in mammalian tissues, body fluids, and gut microbiota [3]. Gut bacterial GUS plays a critical role in hydrolyzing large amounts of endogenous glucuronides and in reactivating various drugs and xenobiotics, including CPT-11 and non-steroidal anti-inflammatory drugs (NSAIDs) [4]. In 2010, Wallace et al. elucidated the crystal structure of *Escherichia coli* GUS (EcGUS) [5], which was shown in Figure 1. The structure comprises two asymmetric subunits, each containing approximately 597 amino acids. The 180 residues at the N-terminus are similar to the sugar-binding domain of GH family 2. Amino acid residues 274–603, located at the C-terminus, form an α/β barrel domain and include the catalytic active residues Glu413 and Glu504. Bacterial loops formed by 17 amino acid residues (360–376) are also present at the C-terminus. The third domain, located between the N- and C-terminus, exhibits an immunoglobulin-like β-sandwich structure.

The discovery and development of effective GUS inhibitors, either synthetic scaffolds or natural products, to ameliorate drug-induced GI toxicity has attracted considerable interest in recent years. Four chemically similar EcGUS inhibitors have been identified using in vitro and cell-based assays [5], and the crystal structures of two of these inhibitors complexed with EcGUS have been determined. Notably, one of the inhibitors alleviated the GI toxicity induced by CPT-11 in vivo. Furthermore, four compounds with distinct chemical structures have been identified as potent and selective EcGUS inhibitors [6]. Wallace et al. designed and synthesized two novel inhibitors (R1 and R3)of EcGUS that did not alter serum CPT-11 levels in mice [7], indicating that these inhibitors did not affect the antitumor activity of CPT-11. Piperazine-containing compounds, including amoxapine and ciprofloxacin, are substrate-dependent inhibitors of GUS that form covalent inhibitor–glucuronide conjugates at the active site of the enzyme [8]. In preclinical models, targeted inhibition of GUS enzymes prevented intestinal toxicity while preserving the antitumor efficacy of CPT-11 [9]. Cheng et al. [4] synthesized a series of pyrazolo[4,3-c]quinolone derivatives and assessed their ability to inhibit GUS. Furthermore, the pharmacokinetic profile of the compound with the strongest inhibitory effect on GUS enzymes was determined [10]. Challa et al. reported the discovery of novel β-GUS inhibitors from chemical libraries using structure-based virtual screening [11] and identified 69 diverse compounds that exhibited positive inhibitory activity in a follow-up in vitro β-GUS biochemical assay.

In addition to synthetic chemical inhibitors, the discovery of potential GUS inhibitors from traditional Chinese medicines and natural products has attracted increasing attention in recent years. A previous study evaluated the inhibitory effects of several natural flavonoids on EcGUS and elucidated their structure–activity relationships [12]. The results indicate that scutellarein and luteolin exhibit strong inhibitory effects on EcGUS. Prenyl flavonoids, sanggenon C, and kuwanon G, derived from mulberries (*Morus alba* L.), were identified as potent broad-spectrum GUS inhibitors through biological evaluations and molecular docking studies [13]. Bai et al. [14] found that amentoflavone, a biflavonoid derived from *Ginkgo biloba*, is also a potent broad-spectrum inhibitor of EcGUS. Furthermore, amentoflavone was identified as the key constituent responsible for the high inhibitory potency of *Selaginella tamariscina* against EcGUS [15]. These studies highlight the significant potential of herbal medicines in the discovery and development of EcGUS inhibitors.

**Figure 2:**
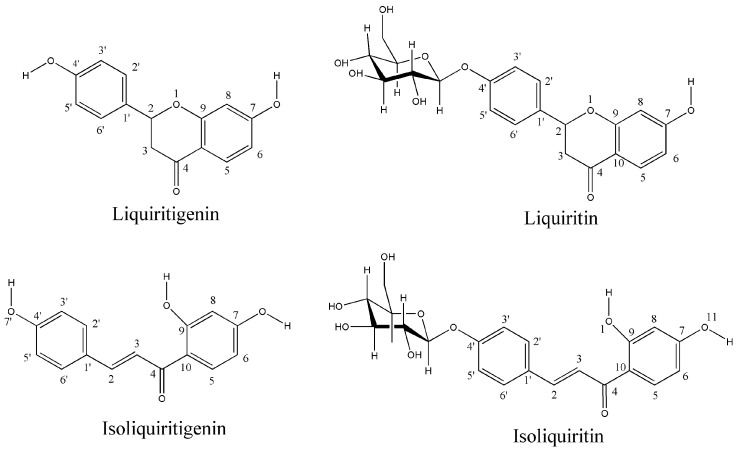
Structures and atom numbering of the four flavonoid compounds derived from licorice.

Licorice is a well-known Chinese herbal medicine first recorded in Shen Nong’s Herbal Classic during the Han Dynasty. It has been extensively used to treat amenorrhea, various forms of cancer, chronic hepatitis, and hyperglycemia [16]. It can also alleviate drug toxicity and detoxify hundreds of drugs [17]. Several studies have investigated the chemical composition and pharmacological activities of licorice [18]. The primary chemical components of licorice are flavonoids and triterpenoid saponins, which exhibit antitumor, anti-inflammatory, antioxidant, antibacterial, and immunomodulatory effects [19]. Our previous research demonstrated that licorice flavonoids reduced the GI toxicity of CPT-11 in vivo [20]. The mechanism by which licorice flavonoids alleviate GI toxicity of CPT-11 was explored using metabolomic methods. Accordingly, we investigated the protective effect of licorice flavonoids on CPT-11-induced colitis in mice from the perspectives of gut microbiota and fecal metabolism [21]. Licorice contains various flavonoids, including liquiritigenin (LG), isoliquiritigenin (ILG), liquiritin (LQ), and isoliquiritin (ILQ). The structures of these four flavonoids are shown in Figure 2. LG and ILG are the aglycones of LQ and ILQ, respectively. The inhibitory effects of LG and ILG on EcGUS were also assessed. The results show that LG and ILG exhibited moderate inhibitory activities against EcGUS, with IC_50_ values of 44.10 ± 1.83 and 32.41 ± 1.61 μM, respectively [22]. This indicates that the inhibitory activity of LG against EcGUS was slightly weaker than that of ILG.

However, the relationship between the structure and the inhibitory activity of licorice flavonoids against EcGUS remains unclear. The mechanism by which the four licorice flavonoids inhibit EcGUS has not yet been investigated in detail, which may hinder their use as lead compounds for developing GUS inhibitors. In this study, a comprehensive computational strategy was employed to investigate licorice flavonoids as potential EcGUS inhibitors. Our study may contribute to the discovery and development of novel GUS inhibitors from Chinese herbal medicine. The computational methods have significant advantages over traditional drug discovery in designing and discovering new drugs. They can improve the efficiency and accuracy of drug discovery and quickly find the most effective drug candidate molecules. These methods can not only help us understand the mechanisms of intermolecular interactions but also narrow down the range of potential candidate compounds before experimental validation, thereby significantly improving the efficiency and success rate of drug development.

## 2. Results and Discussion

### 2.1. Density Functional Theory (DFT) Calculations

From a chemical perspective, the structure of a molecule determines its various properties, including its biological and pharmacological activities [23]. To examine the relationship between the structure of the flavonoids and their inhibition of EcGUS, their structures were fully optimized using the DFT/B3LYP method with the 6-311++G** basis set. The optimized geometrical structures of the four licorice flavonoids (Figure S1) were stable, as indicated by the absence of imaginary frequencies. The calculations reveal that ILG exhibited superior planarity compared to LG, owing to its conjugated structure. Because of its long, conjugated structure, ILG may exhibit a stronger inhibitory effect on EcGUS than LG. In contrast, LQ and ILQ exhibit significant nonplanar structures and steric hindrance owing to the presence of glucose rings. The calculated thermal parameters polarizabilities, and dipole moments are listed in Table 1.

The polarity of a molecule can be evaluated based on the magnitude of its dipole moment. A larger dipole moment indicates higher polarity. The DFT data show that the dipole moments of the studied molecules follow the order: ILG < LG < LQ < ILQ. The dipole moments of these four molecules can help explain their interactions with target proteins. The polarizability of a molecule depends on the proximity of its charges to the electron cloud distribution within the molecular system and is related to the size and complexity of the molecular structure. Larger molecules are more prone to polarization. Notably, the smallest molecule (LG) showed the least polarizability (169.70 Bohr^3^), whereas the molecule with the highest complexity (ILQ) exhibited the highest polarizability (284.45 Bohr^3^).

#### Molecular Electrostatic Potential (MEP)

**Figure 3:**
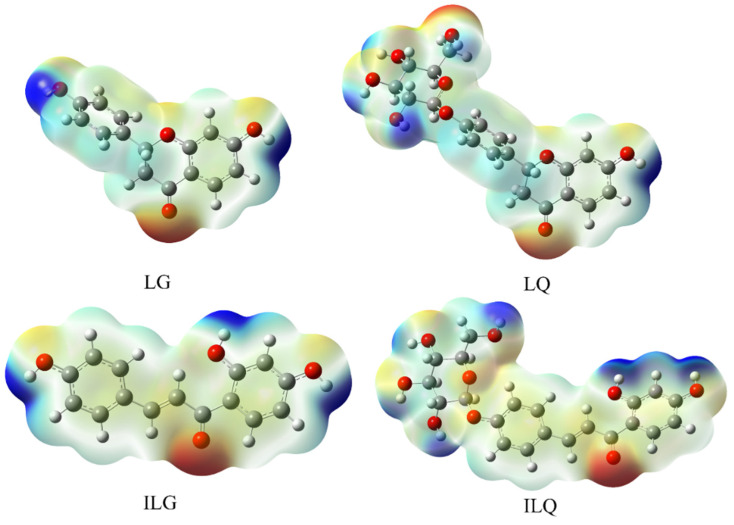
Calculated molecular electrostatic potential surfaces of the optimized structures of LG, LQ, ILG, and ILQ derived from licorice. The positive MEPs (colored in blue), the negative ones (colored in red).

MEP can be used to predict the active binding sites between drug molecules and target proteins, which is crucial for the recognition of ligands and receptors [24]. Variations in the electrostatic potential distribution around the molecules may explain changes in binding affinity with the active site receptor. The MEP calculation results for the four licorice flavonoids are shown in Figure 3. The blue and red regions on the surface correspond to positive and negative MEPs, respectively. For LG and ILG, positive MEPs are concentrated on the hydrogen atoms of the phenolic hydroxyl groups, indicating that this is the most electrophilic site. Negative MEPs are distributed around the carbonyl and hydroxyl oxygen atoms, demonstrating that these oxygen atoms are the most nucleophilic centers. For LQ and ILQ, positive MEPs are distributed not only on the hydrogen atoms of the phenolic hydroxyl groups but also on the hydrogen atoms of the alcohol hydroxyl groups on the glucose ring. Similarly, negative MEPs are located on the oxygen atoms of the alcohol hydroxyl groups attached to the glucose ring. These results indicate that LQ and ILQ may form more interactions with the receptor than LG and ILG. This will be discussed in the following section.

### 2.2. Molecular Docking Study

**Figure 4:**
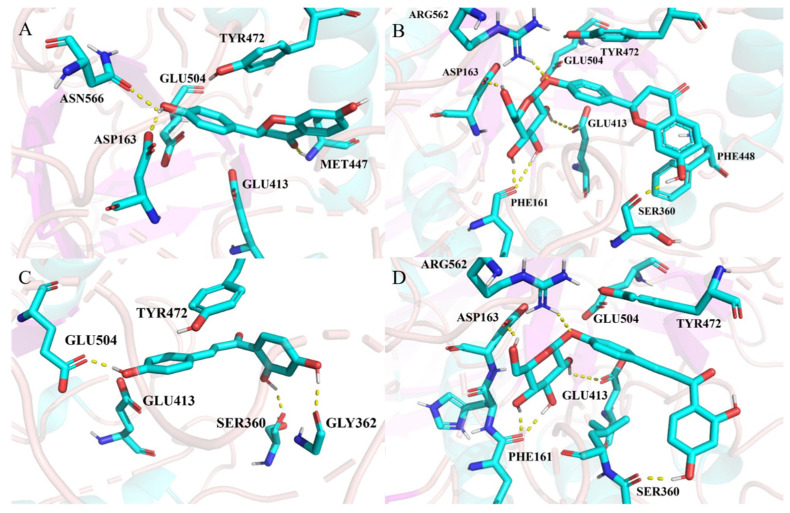
Docking results for the LG-GUS (**A**), LQ-GUS (**B**), ILG-GUS (**C**), and ILQ-GUS (**D**) systems. Ligands and important amino acids are shown as stick diagrams (carbon atoms in cyan, oxygen atoms in red and nitrogen atoms in blue), while EcGUS is represented by a ribbon diagram. Hydrogen bond interactions are indicated by yellow dashed lines. Hydrogen bond is defined as the distance between the acceptor and donor atoms < 3.5 Å, with an internal angle between the H-acceptor and H-donor > 120°.

Molecular docking is a method used to investigate the interactions between small molecules and target proteins and is widely employed in computer-aided drug design [25]. To elucidate the binding mode and mechanism of licorice flavonoids to EcGUS, we conducted a molecular docking study. Figure 4 shows the binding models of the four licorice flavonoids with EcGUS. The structure of EcGUS is different from that of human ortholog due to the presence of 17-residue bacterial loop. This bacterial loop is composed of residues 360 to 376. The active sites of EcGUS consisted of Glu413 (catalytic acid) and Glu504 (catalytic nucleophile).

In the LG-GUS complex, the ligand binds to the catalytic active site of GUS, with the hydroxyl group on the C ring forming hydrogen bonds with Asp163 and Asn566. The carbonyl group on the A ring also forms a hydrogen bond with Met447. In the LQ-GUS complex, the glucose ring on the C ring forms a hydrogen bond network with additional amino acid residues around the active site, including Asp163, Phe161, and Arg562. In particular, the hydroxyl group on the sugar ring forms a hydrogen bond with the catalytic residue Glu413. Notably, the B ring of LQ forms a hydrogen bond with Ser360 in the bacterial loop of GUS. Owing to the presence of a glucose ring, LQ can not only bind to the catalytic active site but also interact with the bacterial loop. This phenomenon resembles that observed in the crystal structure of the EcGUS–inhibitor complex [5], indicating that the bacterial loop is critical for inhibitor efficacy. Therefore, LQ may exhibit a stronger inhibitory effect on GUS than LG.

In the ILG-GUS system, ILG forms hydrogen bonds with the catalytic residue Glu504, as well as with Ser360 and Gly362 in the bacterial loop. The long, conjugated structure of ILG interacts with the bacterial loop of EcGUS. In the ILQ-GUS system, in addition to forming hydrogen bonds with the catalytic residue Glu413, the glucose ring forms additional hydrogen bonds with active amino acids such as Asp163, Phe161, and Arg562. Thus, these findings suggest that the inhibitory effect of ILG on GUS is weaker than that of the glycoside ILQ.

These analyses were based entirely on the docking structures obtained in this study. The stability of each system and the ligand recognition process should be further investigated through long-term molecular dynamics (MD) simulations.

### 2.3. MD Simulation 

**Figure 5:**
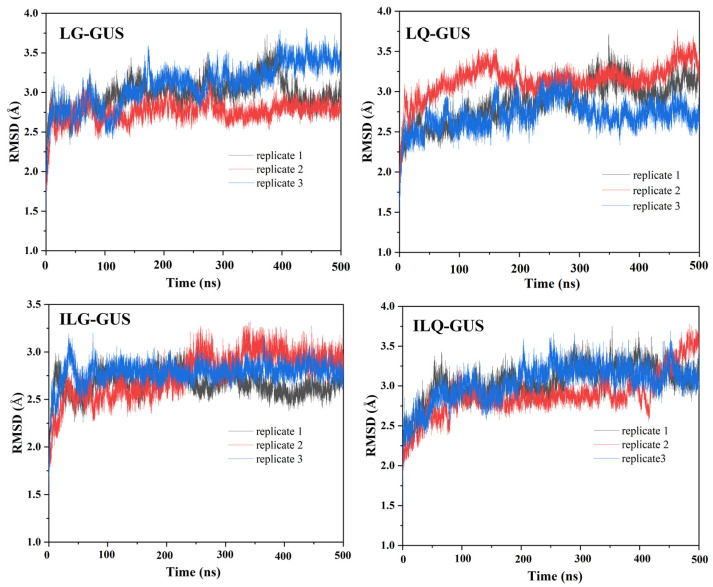
Root mean square deviations (RMSDs) of the heavy atoms of the backbone for the protein receptor EcGUS during 500 ns MD simulations of the LG-GUS, LQ-GUS, ILG-GUS, and ILQ-GUS systems for three replicates.

MD simulation is a computer-aided drug development technique used to design enzyme inhibitors and elucidate the interactions between ligands and receptors [26]. To validate the docking results, 500 ns MD simulations were performed to assess the stability of the four complex systems. The root mean square deviation (RMSD) is an important parameter used to evaluate the stability of a ligand–receptor complex system. We performed three replicates for MD simulations to ensure the reliability of the results. The RMSD values of LG-GUS, LQ-GUS, ILG-GUS, and ILQ-GUS were 0.79 ± 0.14, 2.98 ± 0.19, 2.87 ± 0.25, 2.66 ± 0.12, and 3.04 ± 0.22 Å, respectively, for the first replicate simulation (Table S1). Figure 5 shows the RMSD results obtained from three replicates MD simulations of the four systems. During the 500 ns MD simulations, the RMSDs exhibited minimal fluctuations, indicating stable complex systems. The ligands remained secure in the binding pocket. All four complex structures were sufficiently stable for subsequent analyses.

Because of the different microenvironments of various amino acids, their dynamic equilibrium amplitudes may vary during the simulation process. Root mean square fluctuation (RMSF) values were used to evaluate the dynamics of different amino acids during the simulation process. The RMSF values for the residues were used to identify regions with higher flexibility. As shown in Figure S4, the RMSF distributions in the four complex systems were similar, indicating that the four molecules had similar effects on the conformation of EcGUS. The amino acid residues at positions 13–17, 148–157, 192–200, 230–237, 360–381, and 558–565 of EcGUS exhibited large fluctuations (RMSF > 2.00). Notably, the positions of residues 360–376 fluctuated significantly, demonstrating the flexibility of the bacterial loop. This loop is unique to microbial forms of GUS, in contrast to mammalian GUS [5,27].

The compactness or extensibility of the ligand within the binding pockets during MD simulations can be represented by the radius of gyration. The radii of gyration of the ligands and proteins in these four complex systems are shown in Figure S5. The systems reached a stable state during the simulation. The surfaces of the ligands and proteins in the four complex systems during the 500 ns MD simulations are shown in Figure S6. During the MD simulations, minimal fluctuations were observed on the protein surface within the binding pocket. The surface area of the ligand was small, indicating that it was completely enclosed within the binding pocket. This indicates that most of the solvent-accessible surface of the ligand was surrounded by proteins. Therefore, the ligand can exert inhibitory effects on the proteins to prevent solvent dissolution.

For the four complex systems, conformations were obtained from snapshots at 100, 200, 300, 400, and 500 ns of the MD trajectories and were aligned as shown in Figures S7–S10. Throughout the simulation, the conformation of each ligand remained largely unchanged. Each ligand formed a stable complex with EcGUS in the binding pocket.

The initial docking model determined the existence of a large hydrogen-bonding network between the ligand and the receptor. The hydrogen bonds formed between four ligands and EcGUS were checked. The hydrogen bond occupancy analyses over the MD trajectories for all four binding complexes are shown in Table S2. Only one hydrogen bond with high occupancy (≥90%) was found for ILG binding with EcGUS. The occupancies of hydrogen bonding between the O1 of ILG and the OE1 atom of E504 were 95.82, 94.36, and 96.73% for replicates 1, 2, and 3 of the ILG-GUS systems, respectively.

### 2.4. Binding Free Energy

The inhibitory strength of the inhibitor against the target protein was evaluated by calculating the binding free energy. Tables S3–S6 show the binding free energies of the four complex systems, which were −11.74, −38.11, −20.86, and −45.42 kcal·mol^−1^ for the LG-GUS, LQ-GUS, ILG-GUS, and ILQ-GUS systems, respectively. These results quantitatively demonstrate the inhibitory effects of these four compounds on GUS activity. The predicted order of the inhibitory activity was LG < ILG < LQ < ILQ. The inhibitory effects of the two glycosides (LQ and ILQ) on GUS were stronger than those of their corresponding aglycones (LG and ILG). In contrast to the phenolic group at the C4′ site, the glycosyl group at this position may enhance the inhibitory effects against EcGUS. This suggests that LQ and ILQ are promising candidates for the development pf potential GUS inhibitors. Furthermore, the inhibitory effect of LG on GUS was weaker than that of ILG, which supports the experimental results [22] and demonstrates the significant contribution of the conjugated structure to EcGUS inhibition.

The van der Waals (vdW) interactions for the LG-GUS, LQ-GUS, ILG-GUS, and ILQ-GUS systems were −17.09, −40.08, −23.72, and −43.87 kcal·mol^−1^, respectively. This suggests that vdW interactions facilitate the formation of ligand–receptor complexes. The contributions of the electrostatic interactions to the binding free energies of the four flavonoids were −12.20, −37.25, −40.36, and −64.76 kcal·mol^−1^, respectively. For the LG-GUS and LQ-GUS systems, the contribution of vdW interactions to the binding free energies was markedly greater than that of electrostatic interactions. In contrast, for the ILG-GUS and ILQ-GUS systems, electrostatic interactions contributed more to the binding free energies than vdW interactions.

In general, two main factors affect the binding of inhibitor molecules to proteins: polarity (E_ele_ + E_GB_) and nonpolarity (E_vdw_ + E_surf_). The polar contributions for the LG-GUS, LQ-GUS, ILG-GUS, and ILQ-GUS systems were 7.33, 7.23, 6.57, and 5.36 kcal·mol^−1^, respectively. The positive value of the polar interactions indicates that these interactions were not conducive to binding between the inhibitor and target. Therefore, for these four systems, polar interactions were unfavorable for the inhibition of GUS by the four licorice flavonoids. This implies that a lower energy of the polar interactions corresponds to a stronger inhibitory effect of the small molecule on the receptor. Therefore, among the four flavonoids, ILQ likely exhibits the strongest inhibitory effect on GUS.

For these four complex systems, the total nonpolar values were −19.06, −45.34, −27.43, and −50.78 kcal·mol^−1^, respectively. The negative value of the nonpolar interactions indicates that these interactions promote binding between the inhibitor and the target. This suggests that nonpolar interactions play a significant role in the inhibition of GUS by the four flavonoids. Moreover, these calculations show that the inhibitory effect of the small molecules on GUS can primarily be attributed to their hydrophobic interactions.

As discussed in previous DFT calculations, the negative MEPs of the ligand may facilitate hydrogen-bonding formation or van der Waals interactions with the acceptor. It is evident that the negative electrostatic potential of ILQ is higher than that of other ligands, and this could be an explanation for the lowest binding free energy of ILQ compared with other ligands. Moreover, the dipole moment is another factor that may affect the degree of interaction of these ligands with the protein. ILQ has the highest dipole moment than the others, which could have an effect on the binding free energy.

### 2.5. Free Energy Decomposition

To quantitatively evaluate the contribution of each amino acid residue, the binding energy was decomposed into the interactions between the individual residue and the inhibitor, excluding entropy contributions. The molecular mechanics generalized Born surface area (MM/GBSA) decomposition results are summarized in Tables S7–S10. In particular, several hydrophobic residues contributed significantly to the total binding free energy. For the LG-GUS system, the residue Tyr472 made the largest contribution, suggesting that the π–π stacking interaction between Tyr472 and LG is important for ligand binding. This interaction has also been observed in other flavonoids that bind to EcGUS [28].

For the LQ-GUS system, Asp508 and Tyr472 contributed the most to the subtotal binding free energies. This suggests that these amino acid residues are crucial for the inhibition of EcGUS by LQ. For the ILG-GUS system, residues Tyr472, Glu504, Leu561, and Arg562 in the catalytic active site contributed more than −1.00 kcal·mol^−1^, indicating that these are the key amino acids involved in the ILG inhibition of EcGUS. Notably, Glu504 is a catalytic residue in the active site of EcGUS and is responsible for the hydrolysis of the glucuronide glycoside bond [5].

For the ILQ-GUS system, the catalytic residue Glu413 contributed −2.70 kcal·mol^−1^, owing to its hydrogen-bonding interaction with the alcohol hydroxyl group on the glucose ring. This implies that the alcohol hydroxyl group plays a crucial role in EcGUS binding. Because the catalytic residue Glu413 was conserved in EcGUS [5], the main contributors to ILQ-GUS binding are Leu561, Met447, Ser557, Tyr472, Ser360, Asn358, and Arg562. These residues likely adapted to the ILQ conformation to enhance the interactions. Overall, nonpolar terms, particularly vdW interactions, were the major components of the subtotal binding free energies of most residues. Therefore, the effects of vdW interactions on the binding of hydrophobic residues require special attention.

## 3. Computational Methods

### 3.1. DFT Calculations

The molecular structures of LG, ILG, LQ, and ILQ were optimized using the B3LYP/6-311++G** method. The frequencies of the optimized structures were calculated at the same level, and no imaginary frequencies were found. This implies that the optimized structures represent the best conformations of each compound and can be used as initial conformations for molecular docking. Finally, the thermal parameters and MEP were calculated using the same method. All DFT calculations were performed using the Gaussian 16 software package Revision C.01 from Gaussian Inc. in Wallingford, CT of USA [29].

### 3.2. Molecular Docking

Before conducting molecular docking to build the initial ligand/GUS binding model, we evaluated the docking capability through a “re-docking” strategy. We selected the previously reported EcGUS crystal structure (PDB: 3LPF) [5], which formed a complex with its inhibitor. Based on the docking results, the root mean square deviation (RMSD) between the crystal structure and the conformation with a cluster was 1.6 Å (Figure S2). Therefore, we performed molecular docking to construct the ligand/GUS complex.

The initial structure of EcGUS was extracted from the reported inhibitor–receptor complex structure (PDB ID: 3LPF) [5], which contains a bacterial loop spanning residues 360–376. Proteins and inhibitors were pretreated using AutoDockTools 1.5.6 from Scripps Research Institute of La Jolla, CA, USA [30]. A grid map of 30 × 30 × 30 points with 0.375 Å grid spacing was generated using the AutoGrid 4.2.6 module [31], based on the center of the inhibitor in the EcGUS–inhibitor complex. Docking was performed using the Lamarckian genetic algorithm [32], and the docked compounds underwent 100 runs of the AutoDock search. All docking parameters were consistent across all small molecules. Finally, 100 conformations were constructed for the four systems (LG-GUS, LQ-GUS, ILG-GUS, and ILQ-GUS), and the best conformation from each system was selected for MD simulations.

### 3.3. MD Simulation Method

The four complex systems obtained from the molecular docking study were used as the initial structures for the simulation. The force fields of the small molecules were accurately described using the general Amber force field (GAFF2) generation procedure [33]. The optimized structure of each molecule was determined through DFT calculations. The partial atomic charges of the small molecules were obtained using the restrained electrostatic potential (RESP) protocol [34] in the antechamber module. Topology parameters for EcGUS were generated using the Amber ff19SB force field [35]. All steps were performed using the AMBERTools24 package from University of California in San Francisco of San Francisco, CA, USA [36]. First, the complex systems were solvated using the TIP3P [37] water model. Cl^−^ ions were added based on a Coulomb potential grid to ensure the system remained electrically neutral. The energy of each system was minimized through the following steps to prevent unfavorable interactions between the ions and solvents. First, only the water and counterions were optimized, while the solute molecules were restrained. Second, the entire system was minimized without any constraints. Third, the system was heated from 0 to 300 K in a canonical ensemble (NVT) using Langevin dynamics. The pressure was maintained at 1 bar using an isotropic position-scaling method [38,39]. Subsequently, the system was equilibrated in an NPT ensemble at 300 K and 1 bar. Heating, constant pressure, and equilibration were performed over 200 ps for each process. Finally, 500 ns MD simulations were conducted for each system under the same conditions. In all simulations, the SHAKE algorithm [40] was used to constrain covalent bonds involving hydrogen, and periodic boundary conditions were applied. Long-range electrostatic forces were calculated using the particle mesh Ewald (PME) method [41]. A cut-off of 12 Å was applied for short-range electrostatic forces and vdW interactions. Preparation and analysis were performed using AmberTools24, and simulations were conducted using the CUDA version of the PMEMD in Amber24 [36]. MD trajectory data were analyzed using the CPPTRAJ program [42,43] in Amber24 and AmberTools24.

### 3.4. Binding Free Energy Calculations

The binding free energies between the four molecules and EcGUS were calculated using the MM/GBSA approach [44,45]. This method can efficiently evaluate the binding affinity between a ligand and an enzyme [46,47,48]. The MM/GBSA framework has been extensively described in the literature [49,50,51]. For each complex system, the energy terms were obtained by statistically averaging over 1000 frames of the last 200 ns of the MD trajectory. To assess the contribution of each residue, the total binding energy between EcGUS and the inhibitor was decomposed using MM/GBSA binding free energy decomposition. The contribution of entropy was not considered [52]. The MMPBA.py program [53] in Amber24 and AmberTools24 was used to calculate the energy. All calculations are completed on supercomputer in house and the National Supercomputing Center in Guangzhou. 

## 4. Conclusions

GUS is an important target for alleviating GI toxicity caused by CPT-11. Based on the ability of licorice to mitigate the toxicity of numerous drugs, four licorice flavonoids were evaluated as potential EcGUS inhibitors using DFT, molecular docking, MD simulations, and free energy calculations. DFT results revealed that ILG has a planar structure owing to its conjugation effect. The calculated dipole moments, polarizabilities, and MEPs were used to examine the interactions between the ligand molecule and the acceptor. Among the four flavonoids, ILQ exhibited the highest dipole moment and polarizability.

Molecular docking results indicated that LG binds to the catalytic active site of EcGUS, whereas the other three molecules not only occupy the catalytic sites but also interact with the allosteric sites. The stabilities of these four complex systems were further verified using MD simulations. Furthermore, binding free energy calculations showed that ILQ exhibited the strongest inhibitory effect on EcGUS among the four flavonoids. In summary, our findings revealed the structure–activity relationship and inhibitory mechanism of these four licorice flavonoids against EcGUS at the molecular level. Furthermore, they provide detailed structural information for the discovery and development of effective flavonoid-based EcGUS inhibitors. This study established a theoretical foundation for the potential clinical application of licorice and its flavonoids in alleviating the GI toxicity of CPT-11.

However, the inhibition activity for these potential inhibitors has not been evaluated by an experimental method. This potential clinical application of licorice and its flavonoids should be assayed.

## Figures and Tables

**Table 1:** Thermal parameters (Hartree/particle), polarizabilities (Bohr^3^), and dipole moments (Debye) of liquiritigenin (LG), isoliquiritigenin (ILG), liquiritin (LQ), and isoliquiritin (ILQ).

Parameter	LG	LQ	ILG	ILQ
E_corr_	0.24	0.41	0.23	0.41
ZPVE	−879.50	−1490.08	−879.48	−1490.01
E_tot_	−879.48	−1490.05	−879.46	−1489.98
H	−879.48	−1490.05	−879.46	−1489.98
G	−879.54	−1490.14	−879.53	−1490.07
Total Dipole Moment μ	2.21	4.53	1.96	5.29
Polarizability α	169.70	253.58	198.97	284.45

E_corr_: zero-point correction; ZPVE: sum of electronic and zero-point energies; E_tot_: sum of electronic and thermal energies; H: sum of electronic and thermal enthalpies; G: sum of electronic and thermal free energies.

## Data Availability

The datasets supporting the results of this study are included in the article.

## Supplementary Materials

The following supporting information can be downloaded at: https://www.mdpi.com/article/10.3390/molecules30061324/s1; Figure S1: Optimized geometrical structures of the four flavonoids derived from licorice; Figure S2: The docking result and crystal structure for the inhibitor binding with EcGUS; Figure S3: Root mean square deviations (RMSDs) of the ligand during 500 ns MD simulations; Figure S4: RMSF variations of the C_α_ atom of complexed systems from MD simulations for the (A) LG-GUS, (B) LQ-GUS, (C) ILG-GUS, and (D) ILQ-GUS systems; Figure S5: Radius of gyration from 500 ns MD simulations for the LG-GUS, LQ-GUS, ILG-GUS, and ILQ-GUS systems; Figure S6: Surface areas from 500 ns MD simulations for the LG-GUS, LQ-GUS, ILG-GUS, and ILQ-GUS systems; Figure S7: Snapshots of the LG-GUS system at 100, 200, 300, 400, and 500 ns, along with their aligned forms; Figure S8: Snapshots of the LQ-GUS system at 100, 200, 300, 400, and 500 ns, along with their aligned forms; Figure S9: Snapshots of the ILG-GUS system along the dynamic simulation time for 100, 200, 300, 400, 500 ns and also their aligned form; Figure S10: Snapshots of the ILQ-GUS system along the dynamic simulation time for 100, 200, 300, 400, 500 ns and also their aligned form. Table S1: Root mean square deviation (RMSD*) value of the overall protein of heavy atoms of four complex systems; Table S2: Hydrogen bond analysis between four molecules and EcGUS; Table S3: Binding free energy of LG binding with GUS complexes and decomposition into electrostatic interaction, vdW interaction, and solvation free energies; Table S4: Binding free energy of LQ binding with GUS complexes and decomposition into electrostatic interaction, vdW interaction, and solvation free energies; Table S5: Binding free energy of ILG binding with GUS complexes and decomposition into electrostatic interaction, vdW interaction, and solvation free energies; Table S6: Binding free energy of ILQ binding with GUS complexes and decomposition into electrostatic interaction, vdW interaction, and solvation free energies; Table S7: Free energy decomposition of the LG-GUS complex system at the level of individual residues into contributions from vdW energy, electrostatic interaction energy, nonpolar solvation free energy, polar solvation free energy, backbone energy, and side chain energy; Table S8: Free energy decomposition for the LQ-GUS complex system at the level of individual residues into contributions from vdW energy, electrostatic interaction energy, nonpolar solvation free energy, polar solvation free energy, backbone energy, and side chain energy; Table S9: Free energy decomposition for the ILG-GUS complex system at the level of individual residues into contributions from van der Waals energy, electrostatic interaction energy, nonpolar solvation free energy, polar solvation free energy, backbone energy, and side chain energy. Table S10: Free energy decomposition for the ILQ-GUS complex system at the level of individual residues into contributions from van der Waals energy, electrostatic interaction energy, nonpolar solvation free energy, polar solvation free energy, backbone energy, and side chain energy.
