# Supplementary material for: Natural Flavonoids from Licorice as Potent Inhibitors of β-Glucuronidase Elucidated Through Computational Studies
Source: Molecules. 2025 Mar 15;30(6):1324. doi: 10.3390/molecules30061324 (PMC11945163; doi:10.3390/molecules30061324)
Supplement: Supplementary file 1 [file molecules-30-01324-s001.zip › molecules-3373049-SI.pdf]

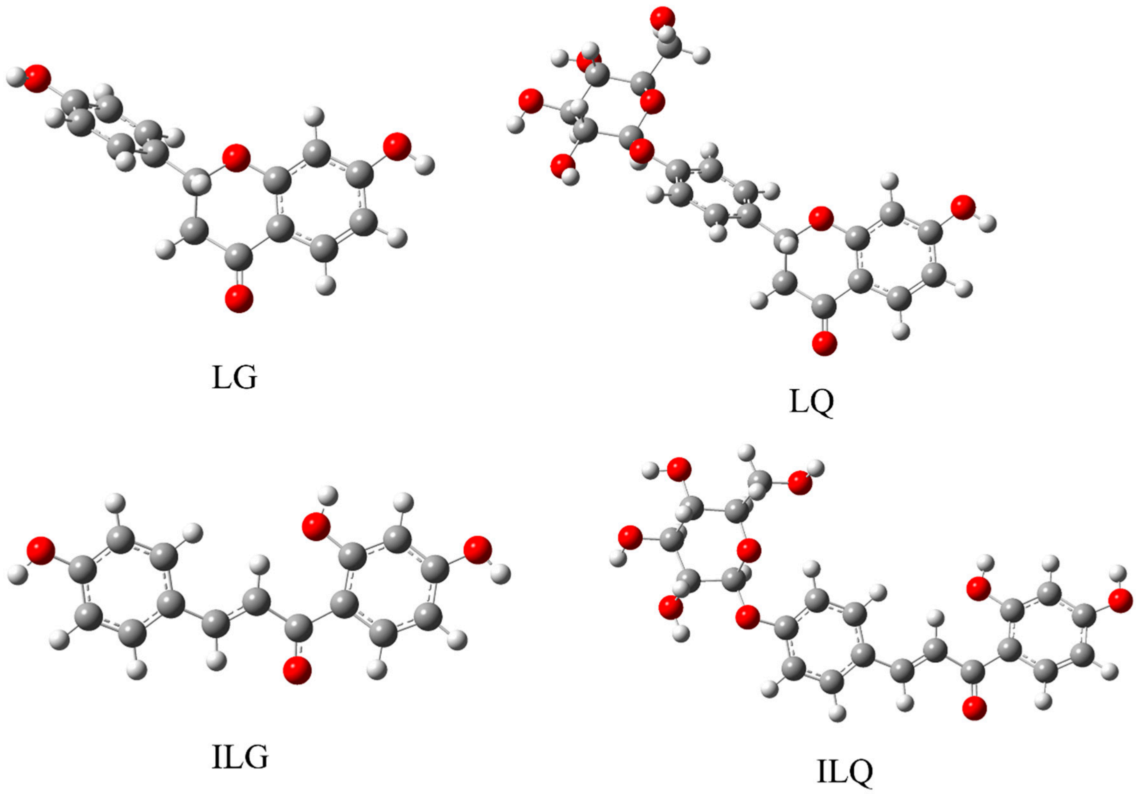

Figure S1. Optimized geometrical structures of the four flavonoids derived from licorice.

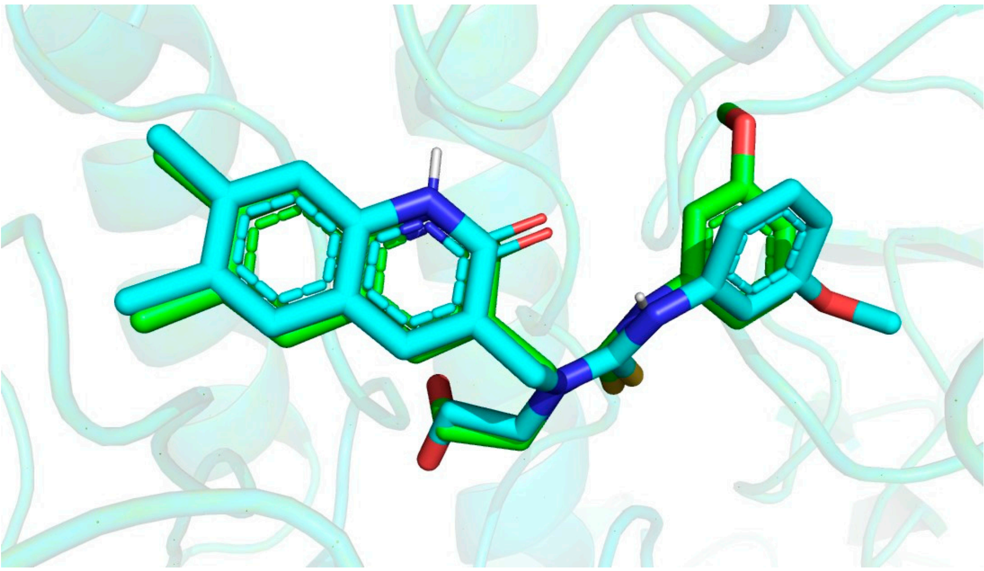

Figure S2. The docking result and crystal structure for the inhibitor binding with EcGUS.Blue for crystal and green for docking result.

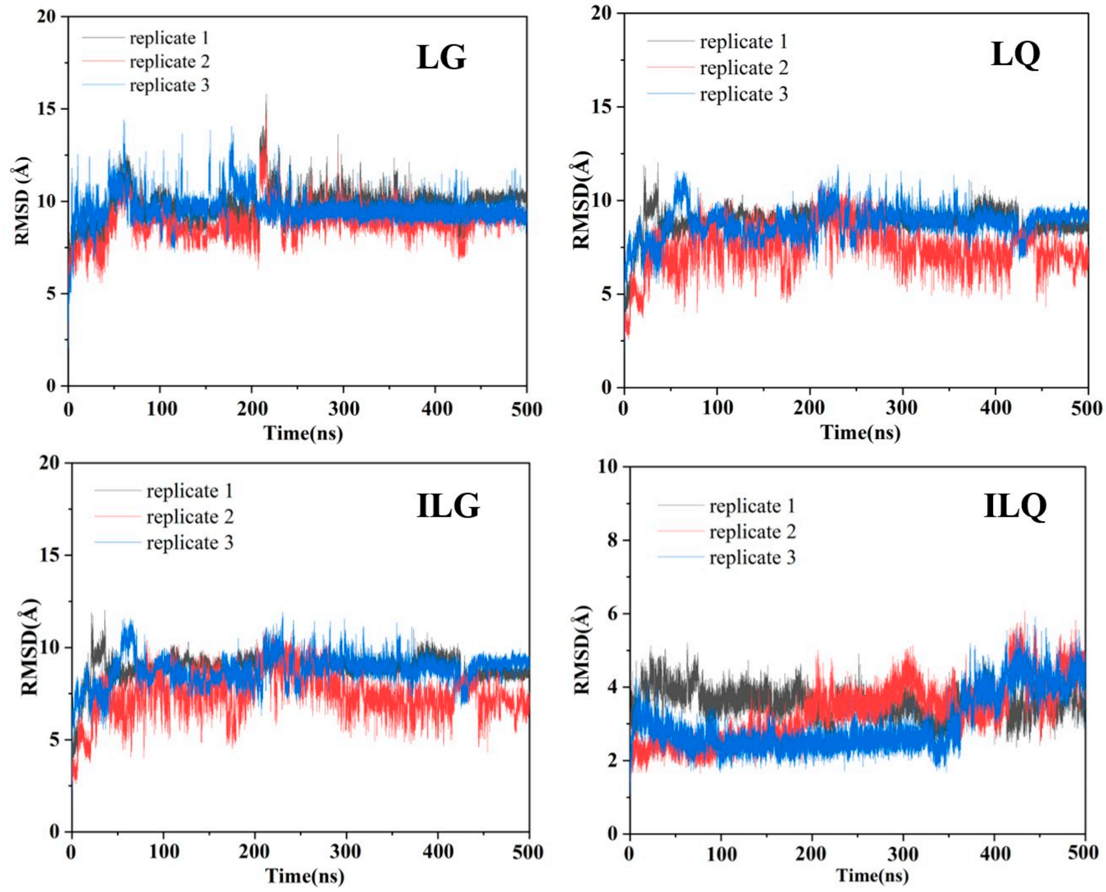

Figure S3. Root mean square deviations (RMSDs) of the ligand during 500 ns MD simulations of the LG, LQ, ILG, and ILQ for three replicates.

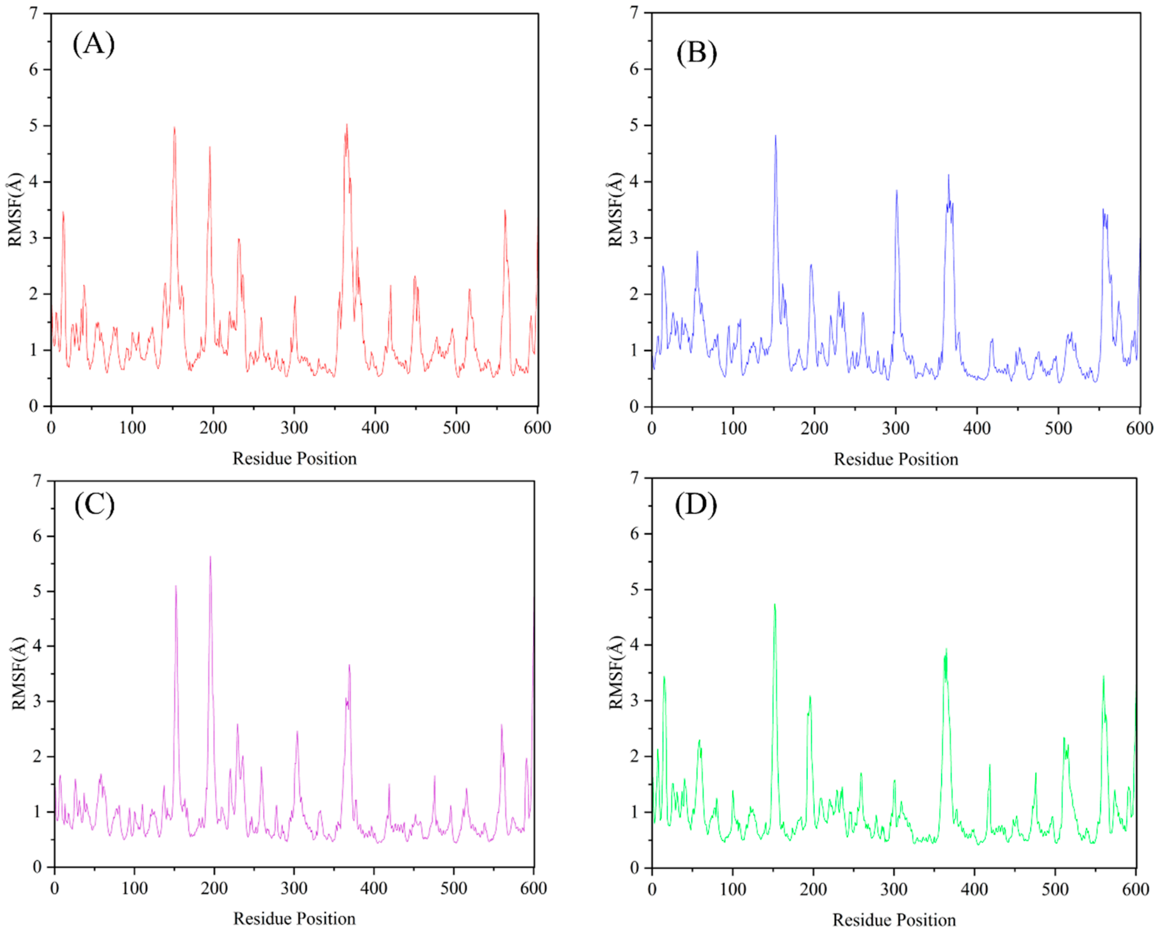

Figure S4. RMSF variations of the Cα atom of complexed systems from MD simulations for the (A) LG-GUS, (B) LQ-GUS, (C) ILG-GUS, and (D) ILQ-GUS systems.

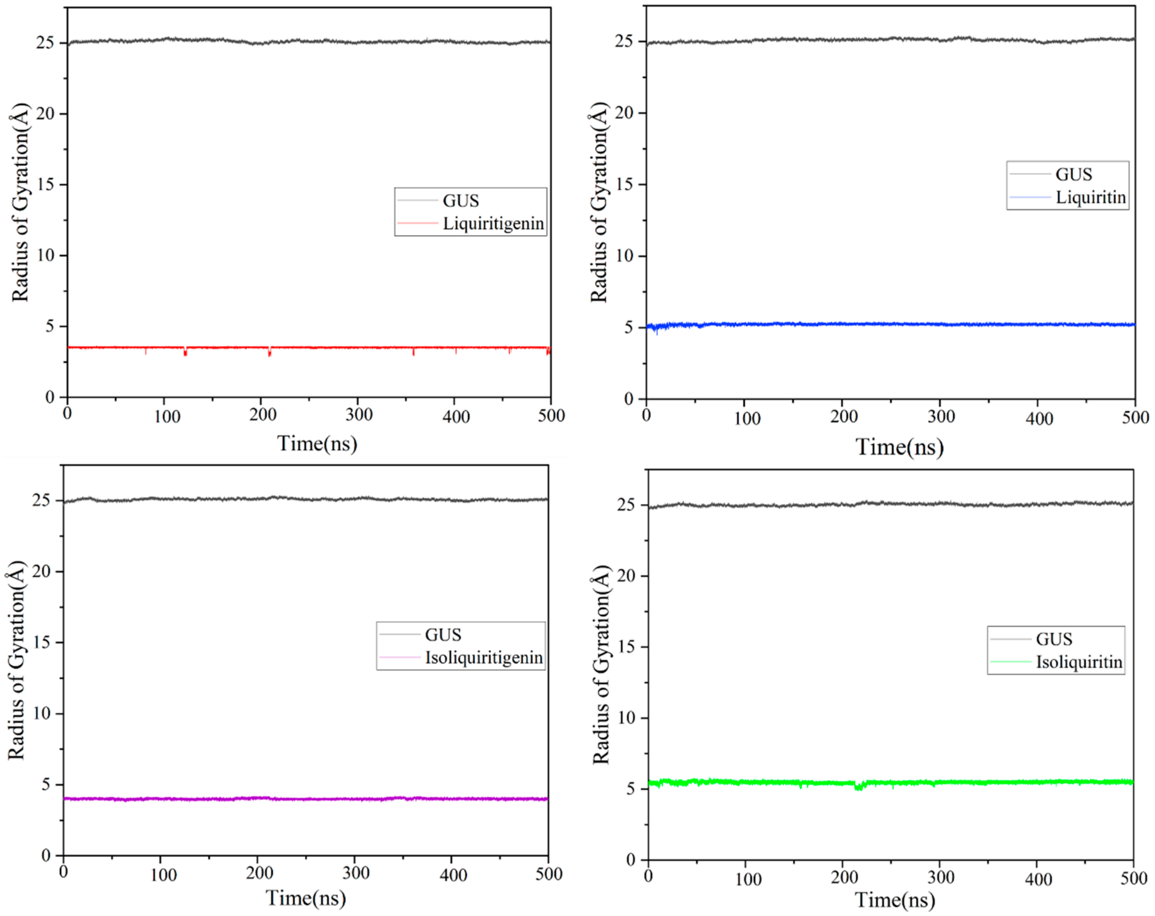

Figure S5. Radius of gyration from 500 ns MD simulations for the LG-GUS, LQ-GUS, ILG-GUS, and ILQ-GUS systems.

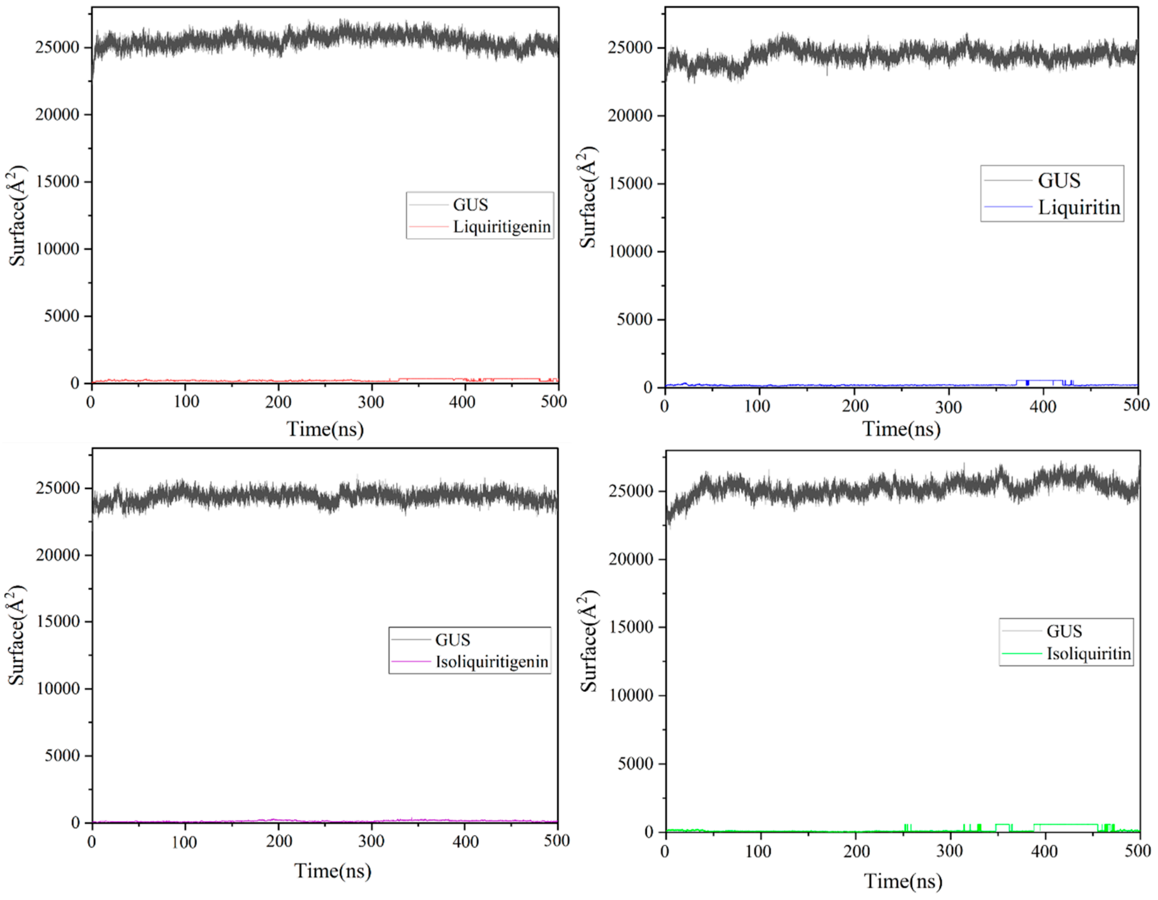

Figure S6. Surface areas from 500 ns MD simulations for the LG-GUS, LQ-GUS, ILG-GUS, and ILQ-GUS systems.

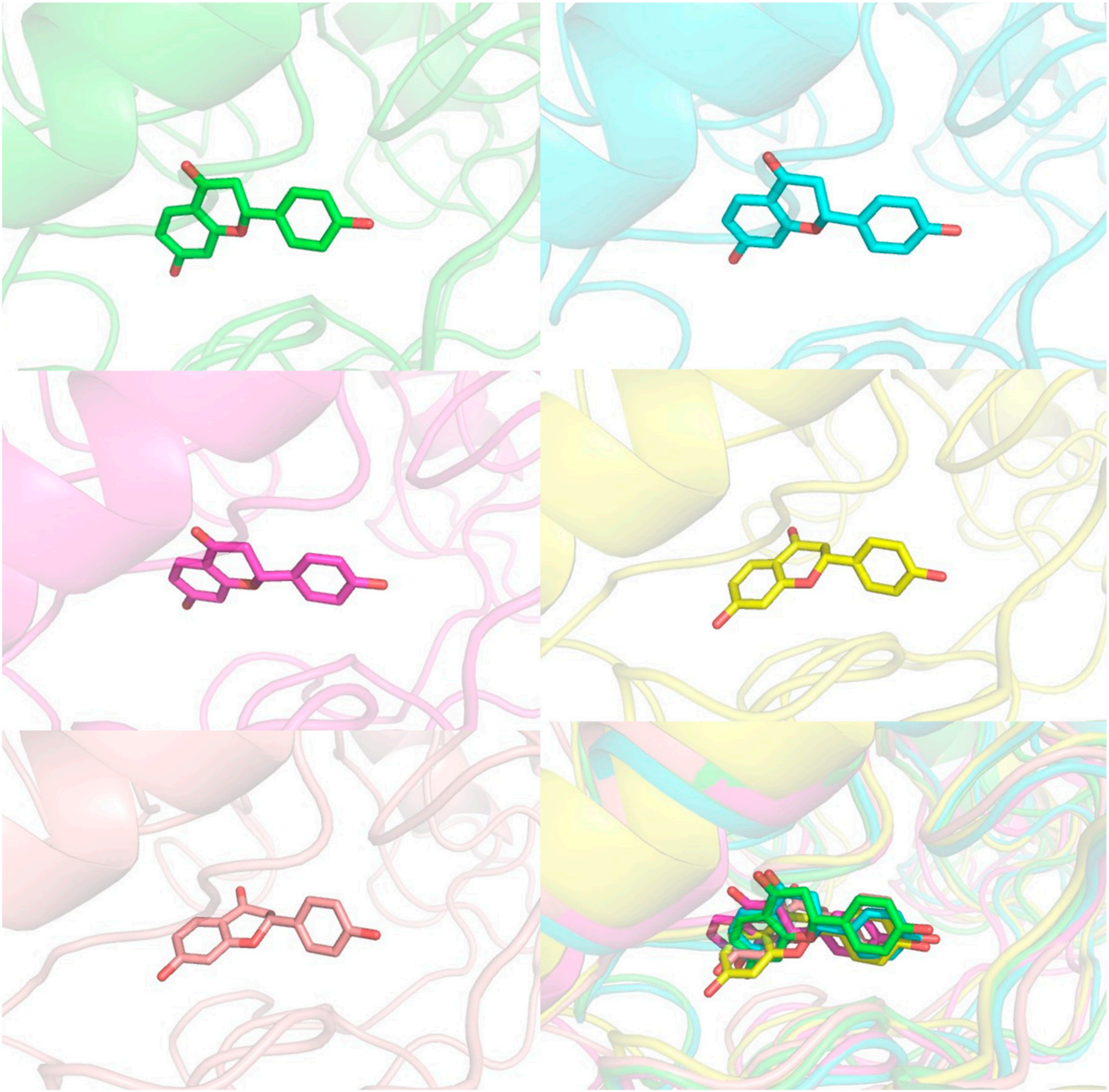

Figure S7. Snapshots of the LG-GUS system at 100, 200, 300, 400, and 500 ns, along with their aligned forms. For clarity, the water molecules have been removed. The inhibitor is plotted using the stick style, while GUS is plotted using the cartoon style.

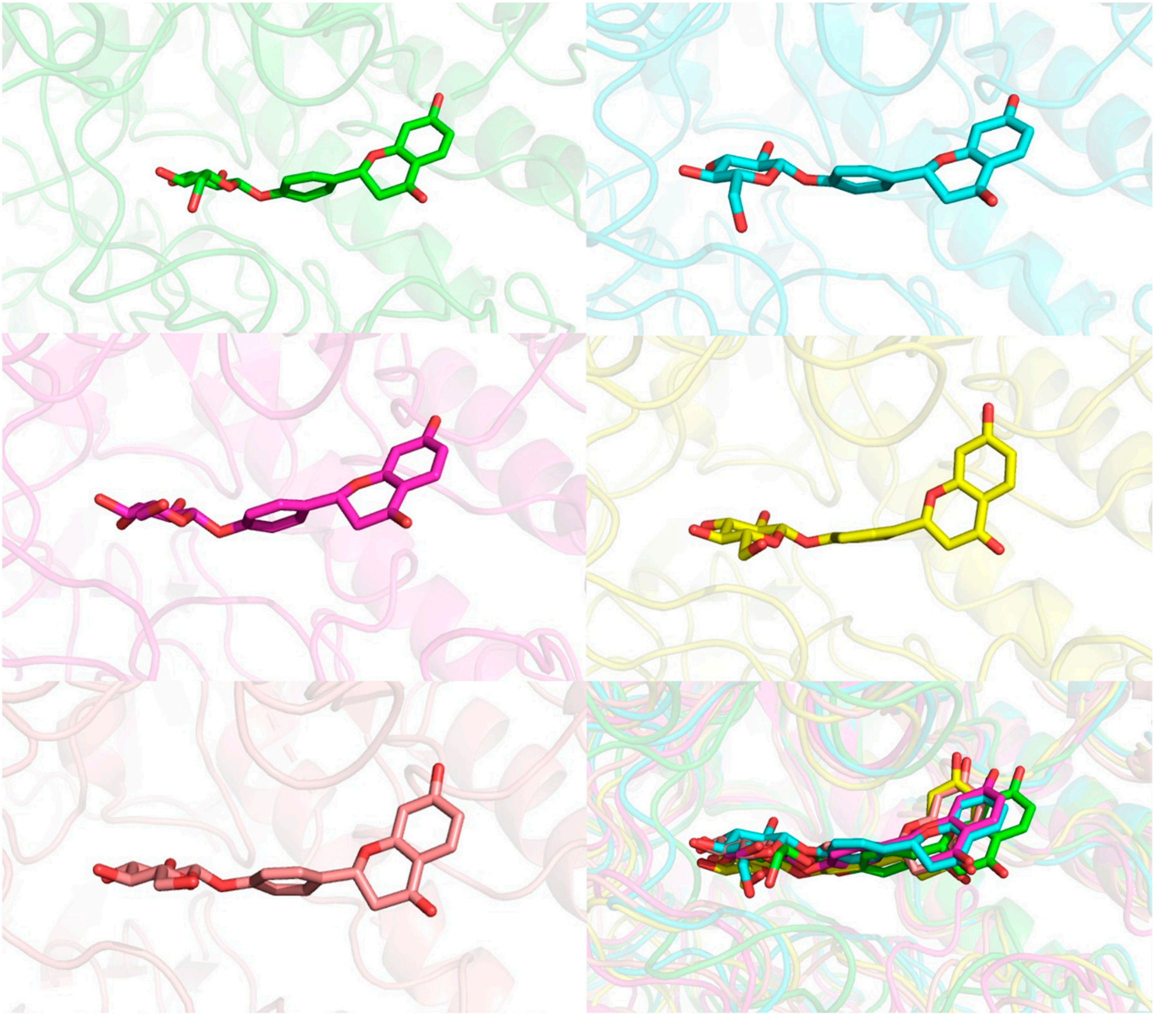

Figure S8. Snapshots of the LQ-GUS system at 100, 200, 300, 400, and 500 ns, along with their aligned forms. For clarity, the water molecules have been removed. Liquiritin is plotted using the stick style, while GUS is plotted using the cartoon style.

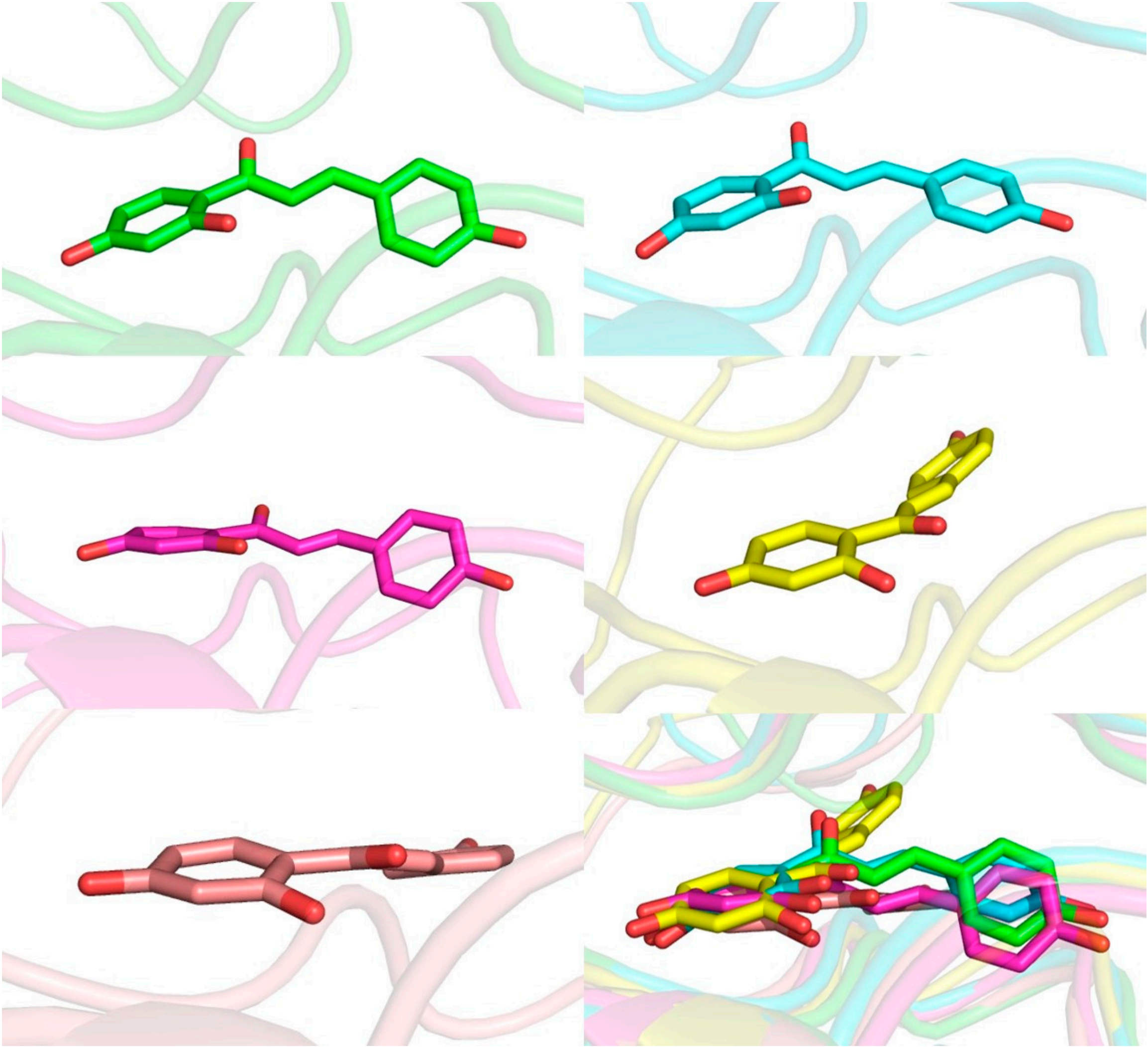

Figure S9. Snapshots of the ILG-GUS system along the dynamic simulation time for 100, 200, 300, 400, 500 ns and also their aligned form. For clarity, the water molecules have been removed. Isoliquiritigenin is plotted using the stick style, while GUS is plotted using the cartoon style.

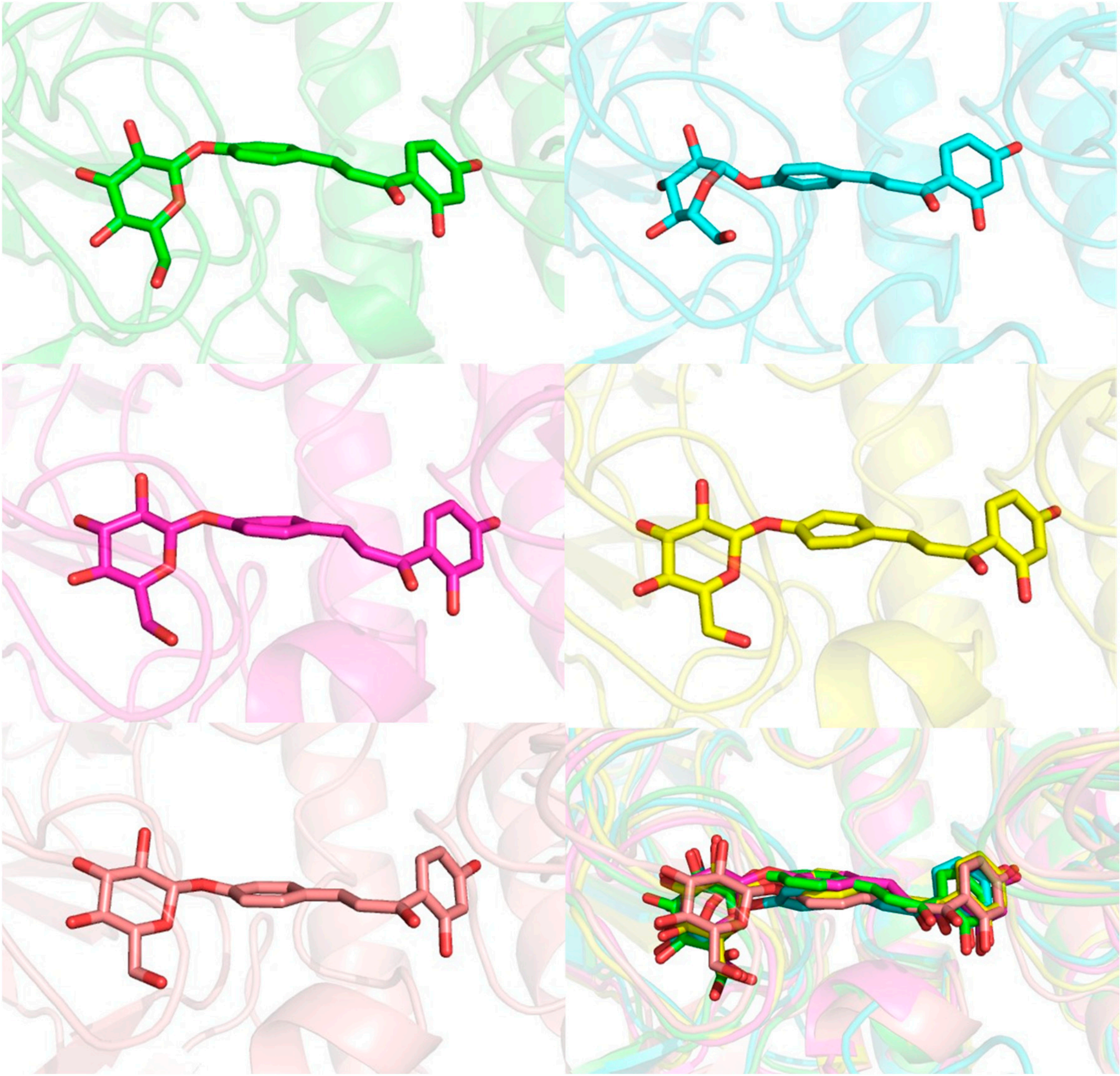

Figure S10. Snapshots of the ILQ-GUS system along the dynamic simulation time for 100, 200, 300, 400, 500 ns and also their aligned form. For clarity, the water molecules have been removed. Isoliquiritin is plotted using the stick style, while GUS is plotted using the cartoon style.

Table S1. Root mean square deviation (RMSD*) value of the overall protein of heavy atoms of four complex systems although 500 ns MD simulations.

		RMSD (Å)	Total RMSD (Å)
LG-GUS	Replicate 1	2.98±0.19	2.94±0.25
	Replicate 2	2.75±0.13	
	Replicate 3	3.09±0.27	
LQ-GUS	Replicate 1	2.87±0.25	2.90±0.28
	Replicate 2	3.14±0.20	
	Replicate 3	2.70±0.18	
ILG-GUS	Replicate 1	2.66±0.12	2.74±0.18
	Replicate 2	2.76±0.24	
	Replicate 3	2.78±0.12	
ILQ-GUS	Replicate 1	3.04±0.22	2.98±0.25
	Replicate 2	2.88±0.27	
	Replicate 3	3.04±0.23	

*Average value ± standard deviation

Table S2. Hydrogen bond analysis between four molecules and EcGUS. The occupancy was expressed as a percentage of the period (500 ns) during which specific hydrogen bonds were formed. The hydrogen bond was confirmed when the distance between the acceptor and donor atoms < 3.5 A, with an internal angle between the H-acceptor and H-donor > 120°

System	Acceptor	Donor	Occupancy (100%)		
			**1**	**2**	**3**
LG-GUS	D163@OD2	LG@O3	67.78	66.26	65.24
LQ-GUS	E504@OE1	LQ@O5	82.80	80.38	81.62
	E413@OE2	LQ@O7	66.45	63.58	64.86
	LQ@O5	Y468@OH	73.49	75.59	76.21
ILG-GUS	E504@OE1	ILG@O1	95.82	94.36	96.73
	S360@O	ILG@O3	72.91	69.89	74.32
ILQ-GUS	E413@OE2	ILQ@O	50.70	53.78	52.45
	D163@OD1	ILQ@O7	67.21	66.78	67.56
	L361@O	ILQ@O1	70.67	69.72	68.98

Table S3. Binding free energy of LG binding with GUS complexes and decomposition into electrostatic interaction, vdW interaction, and solvation free energies.

Energy (kcal/mol)	Complex		Receptor		Ligand		Delta	
	Average	Std. Dev.*	Average	Std. Dev.	Average	Std. Dev.	Average	Std. Dev.
E_{vdW}	-4936.67	30.98	-4917.05	30.66	-2.54	0.38	-17.09	2.11
E_{ele}	-40004.18	122.00	-40063.54	121.59	71.56	1.10	-12.20	5.01
E_{GB}	-9600.00	92.49	-9598.22	92.55	-21.30	0.50	19.53	4.08
E_{surf}	185.05	2.09	184.40	2.03	2.61	0.01	-1.97	0.34
G_{gas}	-8424.74	124.93	-8329.70	124.98	-65.74	3.52	-29.29	5.63
G_{solv}	-9414.95	91.54	-9413.82	91.59	-18.69	0.49	17.56	3.93
$G_{gas} + G_{sol}$	-17839.69	71.34	-17743.52	71.39	-84.43	3.49	-11.74	2.75

* The uncertainties for all of terms are included in the parentheses, which were calculated as the root mean square error for all of frames extracted in the MM/GBSA running.

E_{vdW}: contribution to the free energy of binding from van der Waals energy;

E_{ele}: contribution to the free energy of binding from electrostatic energy;

E_{GB}: contribution to the free energy of binding from polar solvation energies;

E_{surf}: contribution to the free energy of binding from nonpolar solvation energies;

G_{gas}: contribution to the free energy of binding from $E_{vdW} + E_{ele}$;

G_{solv}: contribution to the free energy of binding from $E_{GB} + E_{surf}$.

Table S4. Binding free energy of LQ binding with GUS complexes and decomposition into electrostatic interaction, vdW interaction, and solvation free energies.

Energy (kcal/mol)	Complex		Receptor		Ligand		Delta	
	Average	Std. Dev.*	Average	Std. Dev.	Average	Std. Dev.	Average	Std. Dev.
E_{vdW}	-5031.22	33.49	-4985.97	33.11	-5.17	0.69	-40.08	2.71
E_{ele}	-40390.82	130.31	-40326.67	130.57	-26.90	3.17	-37.25	5.35
E_{GB}	-9300.86	99.50	-9309.76	100.24	-35.59	1.39	44.48	3.73
E_{surf}	171.82	2.29	173.14	2.34	3.94	0.03	-5.26	0.18
G_{gas}	-8719.14	129.76	-8666.05	130.93	24.25	4.61	-77.34	4.68
G_{solv}	-9129.04	98.78	-9136.61	99.48	-31.65	1.38	39.22	3.72
$G_{gas} + G_{sol}$	-17848.18	60.56	-17802.66	60.77	-7.40	4.38	-38.11	2.61

* The uncertainties for all of terms are included in the parentheses, which were calculated as the root mean square error for all of frames extracted in the MM/GBSA running.

E_{vdW}: contribution to the free energy of binding from van der Waals energy;

E_{ele}: contribution to the free energy of binding from electrostatic energy;

E_{GB}: contribution to the free energy of binding from polar solvation energies;

E_{surf}: contribution to the free energy of binding from nonpolar solvation energies;

G_{gas}: contribution to the free energy of binding from $E_{vdW} + E_{ele}$;

G_{solv}: contribution to the free energy of binding from $E_{GB} + E_{surf}$.

Table S5. Binding free energy of ILG binding with GUS complexes and decomposition into electrostatic interaction, vdW interaction, and solvation free energies.

Energy (kcal/mol)	Complex		Receptor		Ligand		Delta	
	Average	Std. Dev.*	Average	Std. Dev.	Average	Std. Dev.	Average	Std. Dev.
E_{vdW}	-4997.23	33.00	-4973.00	32.36	-0.51	1.14	-23.72	3.60
E_{ele}	-40290.61	158.87	-40345.87	158.59	95.62	2.46	-40.36	6.46
E_{GB}	-9233.07	138.36	-9250.00	137.83	-30.00	1.10	46.93	5.15
E_{surf}	176.28	2.94	177.06	2.70	2.93	0.02	-3.71	0.50
G_{gas}	-8823.44	167.04	-8702.99	165.35	-56.37	3.84	-64.08	7.21
G_{solv}	-9056.79	137.07	-9072.94	136.73	-27.06	1.09	43.22	4.99
$G_{gas} + G_{sol}$	-17880.23	73.87	-17775.93	73.20	-83.44	3.75	-20.86	3.55

* The uncertainties for all of terms are included in the parentheses, which were calculated as the root mean square error for all of frames extracted in the MM/GBSA running.

E_{vdW}: contribution to the free energy of binding from van der Waals energy;

E_{ele}: contribution to the free energy of binding from electrostatic energy;

E_{GB}: contribution to the free energy of binding from polar solvation energies;

E_{surf}: contribution to the free energy of binding from nonpolar solvation energies;

G_{gas}: contribution to the free energy of binding from $E_{vdW} + E_{ele}$;

G_{solv}: contribution to the free energy of binding from $E_{GB} + E_{surf}$.

Table S6. Binding free energy of ILQ binding with GUS complexes and decomposition into electrostatic interaction, vdW interaction, and solvation free energies.

Energy (kcal/mol)	Complex		Receptor		Ligand		Delta	
	Average	Std. Dev.*	Average	Std. Dev.	Average	Std. Dev.	Average	Std. Dev.
E_{vdW}	-4953.49	35.01	-4906.62	35.07	-2.98	1.33	-43.87	4.07
E_{ele}	-40019.58	146.74	-39949.18	146.38	-5.64	5.60	-64.76	8.46
E_{GB}	-9630.90	111.39	-9659.97	111.20	-41.05	2.94	70.12	5.35
E_{surf}	183.43	2.92	186.08	2.97	4.26	0.04	-6.91	0.31
G_{gas}	-8312.94	142.20	-8241.67	141.84	37.36	6.81	-108.63	8.19
G_{solv}	-9447.47	110.30	-9473.89	110.11	-36.79	2.93	63.20	5.31
$G_{gas} + G_{sol}$	-17760.42	73.41	-17715.56	73.08	0.57	5.27	-45.42	4.91

* The uncertainties for all of terms are included in the parentheses, which were calculated as the root mean square error for all of frames extracted in the MM/GBSA running.

E_{vdW}: contribution to the free energy of binding from van der Waals energy;

E_{ele}: contribution to the free energy of binding from electrostatic energy;

E_{GB}: contribution to the free energy of binding from polar solvation energies;

E_{surf}: contribution to the free energy of binding from nonpolar solvation energies;

G_{gas}: contribution to the free energy of binding from $E_{vdW} + E_{ele}$;

G_{solv}: contribution to the free energy of binding from $E_{GB} + E_{surf}$.

Table S7. Free energy decomposition of the LG-GUS complex system at the level of individual residues into contributions from vdW energy, electrostatic interaction energy, nonpolar solvation free energy, polar solvation free energy, backbone energy, and side chain energy.

Residue	ΔE_{vdW}	ΔE_{ele}	$\Delta G_{sol,GB}$	$\Delta G_{sol,np}$	$\Delta G_{subtotal}$	$S\Delta G_{subtotal}$	$B\Delta G_{subtotal}$
Trp471	-1.43	-0.84	1.31	-0.16	-1.12	-0.90	-0.23
Tyr472	-3.56	0.23	0.47	-0.51	-3.36	-2.98	-0.38
Leu561	-1.31	0.41	-0.04	-0.36	-1.30	-1.37	0.07
Lys568	-0.07	-6.33	5.49	-0.09	-1.00	-1.06	0.06

Energies are in kcal/mol.

ΔE_{vdW}: contributions from van der Waals energy;

ΔE_{ele}: contributions from electrostatic interaction energy;

$\Delta G_{sol,GB}$: contributions from polar solvation free energy;

$\Delta G_{sol,np}$: contributions from nonpolar solvation free energy;

$\Delta G_{subtotal}$: contributions from binding free energy;

$S\Delta G_{subtotal}$: contributions from side chain energy;

$B\Delta G_{subtotal}$: contributions from backbone energy.

Table S8. Free energy decomposition for the LQ-GUS complex system at the level of individual residues into contributions from vdW energy, electrostatic interaction energy, nonpolar solvation free energy, polar solvation free energy, backbone energy, and side chain energy.

Residue	ΔE_{vdW}	ΔE_{ele}	$\Delta G_{sol,GB}$	$\Delta G_{sol,np}$	$\Delta G_{subtotal}$	$S\Delta G_{subtotal}$	$B\Delta G_{subtotal}$
Asp508	0.05	-7.92	4.05	-0.01	-3.83	-3.82	-0.01
Tyr472	-4.66	-0.58	2.30	-0.49	-3.43	-2.80	-0.63
Val563	-1.35	-3.57	2.54	-0.21	-2.60	-1.05	-1.55
Leu561	-2.18	-0.11	0.97	-0.40	-1.71	-1.53	-0.18
Trp471	-1.87	-0.66	1.09	-0.26	-1.70	-1.52	-0.18
Val473	-1.59	0.03	0.24	-0.29	-1.62	-1.58	-0.04
Met447	-1.87	0.32	0.40	-0.25	-1.41	-1.23	-0.18
Phe448	-1.50	-0.17	0.54	-0.27	-1.40	-1.19	-0.21
Arg562	-1.45	-1.53	2.24	-0.06	-0.80	-0.76	-0.04

Energies are in kcal/mol.

ΔE_{vdW}: contributions from van der Waals energy;

ΔE_{ele}: contributions from electrostatic interaction energy;

$\Delta G_{sol,GB}$: contributions from polar solvation free energy;

$\Delta G_{sol,np}$: contributions from nonpolar solvation free energy;

$\Delta G_{subtotal}$: contributions from binding free energy;

$S\Delta G_{subtotal}$: contributions from side chain energy;

$B\Delta G_{subtotal}$: contributions from backbone energy.

Table S9. Free energy decomposition for the ILG-GUS complex system at the level of individual residues into contributions from van der Waals energy, electrostatic interaction energy, nonpolar solvation free energy, polar solvation free energy, backbone energy, and side chain energy.

Residue	ΔE_{vdW}	ΔE_{ele}	$\Delta G_{sol,GB}$	$\Delta G_{sol,n}$	$\Delta G_{subtotal}$	$S\Delta G_{subtotal}$	$B\Delta G_{subtotal}$
Tyr472	-3.04	-0.01	1.21	-0.37	-2.21	-2.14	-0.01
Glu504	0.62	-13.78	11.40	-0.09	-1.85	-1.85	0.00
Leu561	-1.41	-1.18	1.20	-0.23	-1.62	-1.32	0.00
Arg562	-1.30	-3.50	3.88	-0.24	-1.17	-1.01	0.00
Met447	-0.77	-0.03	0.09	-0.14	-0.84	-0.79	0.00
Ile560	-0.66	-0.50	0.52	-0.15	-0.78	-0.66	0.00
Lys568	-0.78	-1.33	1.51	-0.11	-0.72	-0.71	0.00
Phe448	-0.74	-0.16	0.48	-0.23	-0.65	-0.68	0.00

Energies are in kcal/mol.

ΔE_{vdW}: contributions from van der Waals energy;

ΔE_{ele}: contributions from electrostatic interaction energy;

$\Delta G_{sol,GB}$: contributions from polar solvation free energy;

$\Delta G_{sol,np}$: contributions from nonpolar solvation free energy;

$\Delta G_{subtotal}$: contributions from binding free energy;

$S\Delta G_{subtotal}$: contributions from side chain energy;

$B\Delta G_{subtotal}$: contributions from backbone energy.

Table S10. Free energy decomposition for the ILQ-GUS complex system at the level of individual residues into contributions from van der Waals energy, electrostatic interaction energy, nonpolar solvation free energy, polar solvation free energy, backbone energy, and side chain energy.

Residue	ΔE_{vdW}	ΔE_{ele}	$\Delta G_{sol,GB}$	$\Delta G_{sol,np}$	$\Delta G_{subtotal}$	$S\Delta G_{subtotal}$	$B\Delta G_{subtotal}$
Glu413	0.48	-12.78	9.67	-0.06	-2.70	-2.66	-0.04
Leu561	-2.12	-2.02	1.99	-0.30	-2.45	-1.81	-0.64
Met447	-1.43	-2.09	1.66	-0.13	-1.99	-1.38	-0.62
Ser557	-0.51	-2.71	1.61	-0.13	-1.75	-0.33	-1.42
Tyr472	-2.23	0.57	0.26	-0.33	-1.73	-1.66	-0.07
Ser360	-1.51	-1.34	1.51	-0.27	-1.61	-1.53	-0.09
Asn358	-1.10	0.00	-0.21	-0.11	-1.41	-1.31	-0.10
Arg562	-2.66	-0.67	2.18	-0.22	-1.36	-1.42	0.06
Val446	-0.62	-0.59	-0.03	0.00	-1.24	-0.47	-0.77
Ile560	-0.83	-0.40	0.25	-0.04	-1.02	-0.47	-0.55
Thr556	-0.83	-0.07	0.04	-0.06	-0.92	-0.88	-0.04
Leu361	-0.85	-0.04	0.29	-0.16	-0.77	-0.77	0.01
Tyr468	-0.89	0.38	0.03	-0.07	-0.55	-0.56	0.01

Energies are in kcal/mol.

ΔE_{vdW}: contributions from van der Waals energy;

ΔE_{ele}: contributions from electrostatic interaction energy;

$\Delta G_{sol,GB}$: contributions from polar solvation free energy;

$\Delta G_{sol,np}$: contributions from nonpolar solvation free energy;

$\Delta G_{subtotal}$: contributions from binding free energy;

$S\Delta G_{subtotal}$: contributions from side chain energy;

$B\Delta G_{subtotal}$: contributions from backbone energy.
